# PSMA-Targeting Positron Emission Agents for Imaging Solid Tumors Other Than Non-Prostate Carcinoma: A Systematic Review

**DOI:** 10.3390/ijms20194886

**Published:** 2019-10-02

**Authors:** Christophe Van de Wiele, Mike Sathekge, Bart de Spiegeleer, Pieter Jan de Jonghe, Laurence Beels, Alex Maes

**Affiliations:** 1 Department of Nuclear Medicine, AZ Groeninge, 8500 Kortrijk, Belgium; Pieterjan.DeJonghe@azgroeninge.be (P.J.d.J.); Laurence.Beels@azgroeninge.be (L.B.); alex.maes@azgroeninge.be (A.M.); 2 Department of Nuclear Medicine and Radiology, University Ghent, 9000 GHent, Belgium; 3 Department of Nuclear Medicine, University of Pretoria, 0001 Pretoria, South-Africa; mike.sathekge@up.ac.za; 4 Laboratory of Drug Quality and Registration, University Ghent, 9000 Ghent, Belgium; bart.despiegeleer@ugent.be; 5 Department of Imaging and Pathology, KULAK, University of Leuven, 3000 Leuven, Belgium

**Keywords:** PSMA, positron emission tomography (PET), non prostate carcinoma

## Abstract

Despite its name, prostate-specific membrane antigen (PSMA) has been shown using immunohistochemistry (IHC) to also be over-expressed in the tumor neovasculature of a wide variety of solid tumors other than prostate carcinoma. Accordingly, positron-emitting radiolabeled small molecules targeting PSMA, initially developed for positron emission tomography in prostate carcinomas, are currently being explored for their staging and restaging potential as an alternative imaging modality in other solid tumor types where 18-F-fluorodeoxyglucose (FDG)-PET imaging has low diagnostic accuracy. In this paper, the currently available literature in this field is reviewed. Preliminary, mainly retrospective studies are encouraging, with evidence of improved diagnostic sensitivity and specificity in clear cell renal carcinoma, glioma, and hepatocellular carcinoma, leading to a change in patient management in several patients. However, the results published thus far warrant confirmation by larger prospective studies additionally assessing the longitudinal impact on patient outcomes.

## 1. Introduction

Prostate-specific membrane antigen (PSMA), an integral membrane glycoprotein ectopeptidase with both folate hydrolase and N-acylated, a-linked dipeptidase activities, was first characterized in 1986 by the murine monoclonal antibody 7E11, derived from mice immunized with partially purified cell membrane fractions isolated from the human prostate adenocarcinoma cell line LNCap [1,2,3]. Subsequent immunohistochemical (IHC) analysis showed PSMA to be highly expressed in the epithelial cells of the prostate with an intense over-expression in prostate cancer, where its increased expression was shown to correlate with advanced disease stage and the presence of distant metastases [2,4,5]. In recent years, PSMA-targeting positron emission tomography (PET) imaging, primarily using the radiolabeled urea-based small molecular PSMA inhibitors 68Ga-PSMA-HBED-CC and 18F-DCFPyL, has emerged as an important modality for imaging prostate carcinomas with promising clinical results, leading to an increase in the number of studies exploring the role of PSMA PET in detection, staging, restaging at biochemical recurrence, prognostication, and treatment response assessment of prostate carcinoma [5]. In addition, limited available data suggest that alpha- and beta-emitting PSMA-targeting derivatives are highly promising for the treatment of prostate cancer, and rapid development of these agents is anticipated in the years ahead assuming adequate isotope availability and appropriate clinical trials [5].

Aside from its overexpression on epithelial cells of prostate carcinomas, immunohistochemistry studies have shown that PSMA is also upregulated on the endothelial cells of the neovasculature of a wide variety of other solid tumors. Accordingly, in patients suffering from these tumor types, PET imaging using PSMA-targeting radiolabeled tracers may prove of interest for the staging, restaging, and identification of patients that might potentially benefit from PSMA-targeting treatment strategies. In this paper, the currently available literature in this field is reviewed. Only studies describing results in at least three patients are included.

## 2. Renal Cell Carcinoma

Renal cell carcinoma (RCC) is a collection of different types of tumors with distinct genetic characteristics, histological features, and, to some extent, clinical phenotypes [6]. The most common types of renal cell carcinoma are the proximal-convoluted-tubule-derived clear cell renal carcinoma (CRCC), accounting for up to 75% of renal cell carcinomas; the papillary renal cell carcinoma (PRCC), accounting for another 15% of renal cell carcinomas; and the collecting-tubule-derived chromophobe renal cell carcinoma (ChRCC), accounting for 5% of renal cell carcinomas and oncocytomas. As diagnosis of RCC is frequently not made until disease is either locally advanced and unresectable or metastatic, and as many patients who initially present with resectable disease eventually recur, the overall survival of patients suffering from RCC is poor; the estimated five-year survival rate is only 50% [6,7,8]. While CT and MRI imaging are routinely used for staging RCC, particularly in small lesions and subcentimeter lymph nodes potentially representing early metastases, definitive diagnosis remains difficult using these imaging modalities [9]. Furthermore, the role of bone scintigraphy and 18-F-fluorodeoxyglucose (FDG) PET/CT imaging in the staging of RCC is limited. As a consequence, a subgroup of patients with limited metastatic burden, termed oligo-metastatic disease, may be deprived of long-term disease control or cure. In the search for novel, clinical, useful molecular-targeted imaging biomarkers that may improve staging and grading and thus also hopefully improve outcome in RCC patients, a number of authors have reported on 68Ga-PSMA-PET CT imaging results in RCC patients.

68GA-PSMA-HBED-CC PET imaging: Rhee et al. prospectively performed 68Ga-PSMA-HBED-CC PET/CT imaging within 4 weeks following standard imaging (CT of the chest and abdomen and, in some cases, additionally ultrasound, bone scan, or MRI) in ten patients with newly diagnosed renal tumor (eight CRCC, one PRCC, one unclassified) and suspicion of metastatic disease on standard imaging according to RECIST 1.1 criteria (measurable lesions defined as lymph nodes greater than or equal to 10 mm in the short axis, or tumor lesions with minimum size of 10 mm by CT scan or 20 mm by chest X-ray) [10]. Of the 86 PET abnormalities reported as primary or metastatic disease, histological correlation was available in 36, out of which 35 proved to harbor renal cell carcinoma deposits. Inversely, out of 32 CT-identified lesions that were surgically removed or biopsied for histopathological correlation, only 24 were consistent with RCC. The greatest advantage of 68Ga-PSMA PET/CT imaging over standard CT proved to be its ability to identify small lesions (the smallest node identified was 6 mm) or lesions in areas where visualization is difficult, such as in the liver, especially when contrast cannot be used. 68Ga-PSMA HBED-CC PET imaging led to the alteration of patient management in two patients: in one patient, 68Ga-PSMA-HBED-CC PET imaging identified a small liver metastasis that was not revealed on non-contrast MRI, US, or CT, whereas in another patient, 68Ga-PSMA-HBED-CC PET imaging identified a tumor thrombus extension into a lumbar vein which was excised using the PET image as a guide. Of interest, the SUVmax of the PRCC proved 6 times lower than the average SUVmax of the remaining tumors. Sawicki et al. retrospectively reviewed 68Ga-PSMA-HBED-CC PET imaging results obtained in six RCC patients (four CRCC, one PCRCC, and one chromophobe RCC) [11]. 68Ga-PSMA-HEBD-CC PET/CT imaging identified 22 lesions: 5 primary RCCs, 16 metastases (all of which were found in two CRCC patients), and 1 benign lesion which was consistent with ectopic salivary gland tissue in the left masseter muscle. Eight of the 16 metastases displayed focal 68Ga-PSMA-HBED-CC uptake: 2 pleural metastases, 3 bone metastases, and 3 lung metastases (size range 1.0–1.7 cm). The remaining eight PET-negative metastases were all lung metastases identified on CT with size ranging from 0.5 to 0.9 cm. Furthermore, visualization of the primary RCC was not feasible as none of the primary tumors showed 68GaPSMA-HBED-CC uptake above that of the surrounding renal parenchyma. The authors concluded that 68Ga-PSMA HEBD-CC PET/CT does not offer additional value in the local staging of RCC when compared to CT imaging. Inversely, in a recent retrospective study by Raveenthiran et al., patient management as defined based on diagnostic CT findings was changed in 8 out of 16 RCC patients (8 CRCC, 1 PRCC, 1 oncocytoma, 6 unknown histology) following subsequent 68Ga-PSMA HEBD-CC PET/CT imaging; 68Ga-PSMA-HBED-CC PET imaging identified new metastatic disease in two patients not present on standard CT imaging, refuted disease in involved lymh nodes as defined by diagnostic CT imaging in three patients (stable on restaging), and identified new synchronous primaries (two prostate and one lung carcinoma) in three patients [12]. Likewise, in 22 RCC patients (20 CRCC patients, 1 CRCC patient, and 1 transitional cell carcinoma), patient management as defined based on initial diagnostic CT findings was changed in 13 out of 20 patients following subsequent 68Ga-PSMA HEBD-CC PET/CT imaging performed at restaging: in 4/9 patients, new sites of metastatic disease were identified; in 5/9 patients, 68Ga-PSMA-HBED-CC PET/CT imaging refuted disease; and in another 4 patients, a synchronous primary prostate carcinoma was identified. Finally, Siva et al. reported on the diagnostic value of 68GaPSMA-HBED-CC PET imaging compared to conventional imaging as well as FDG PET/CT imaging in a retrospective series of eight RCC patients (seven CRCC and one PRCC) [13]. In all but two cases—the sole PRCC and one CRCC—FDG PET and 68Ga-PSMA-HEBD-CC PET findings proved concordant in the detection of the site of disease. In one of the latter two patients, two additional lesions were detected on 68GA-PSMA-HBED-CC PET imaging, resulting in a change in treatment planning (from stereotactic ablative radiotherapy to systemic therapy). Of interest, both PET techniques demonstrated response to therapy earlier than CT or MRI imaging.

18F-DCFPyL PET/CT imaging: In a series of five patients by Rowe et al., 18 lesions suspicious for CRCC were found on conventional imaging versus 28 on 18F-DCFPyL PET, 17 of which corresponded to sites of disease on conventional imaging [14]. The only lesion that PET was unable to identify was a 6 mm liver lesion. In contrast, PET identified small lymph nodes in the mediastinum or retroperitoneum as well as occult bone metastases that were not identified on conventional imaging. Meyer et al. prospectively performed 18F-DCFPyL PET/CT imaging in 14 patients diagnosed with oligo-metastatic CRCC based on standard-of-care conventional cross-sectional imaging with CT and/or MRI. In 4 out of 14 patients, 18F-DCFPyL PET/CT imaging identified a total of 12 additional lesions, resulting in 3 patients being considered as no longer suffering from oligo-metastatic but from more widespread disease [15]. Additionally, in one patient who still met the criteria for oligo-metastatic disease, new sites of disease were identified. Finally, Yin et al. performed 18F-DCFPyL PET/CT imaging in eight patients suffering from metastatic non-clear cell RCC (three PRCC, two chromophobe RCC, two unclassified RCCs, and one Xp11 translocation RCC) [16]. Of the 73 putative sites of metastatic disease and 3 primary renal lesions identified on the basis of conventional imaging, no lesions were identified on PET/CT imaging without a corresponding finding on conventional imaging, and only 10 of the 73 suspected lesions had definitive uptake of 18F-DCFPyL PET/CT imaging. An additional 14 lesions showed equivocal uptake, and the remaining 49 lesions showed no significant tracer uptake above the background activity.

## 3. Transitional Cell Carcinoma of the Bladder

Bladder tumors, 90% of which are derived from the urothelium, are characterized by a high recurrence rate and an aggressive clinical course [17,18,19]. Parameters predicting bladder carcinoma progression and survival include the depth of tumor infiltration, the histologic differentiation grade, and the presence of concomitant carcinoma in situ. While 80% of transitional cell carcinomas of the bladder are initially superficial, 30%–90% of these will develop a local recurrence following localized therapy, of which 15%–20% will evolve to invasive and/or metastatic stages following retreatment (local, systemic, intra-vesical) [20]. Accordingly, any biological marker capable of selecting those patients that are at risk of progression and who may thus benefit from earlier aggressive treatment is of clinical interest. Campbell et al. performed 18F-DCFPyL PET/CT imaging in three patients suffering from metastasized urothelial carcinoma [21]. While 18F-DCFPyL PET/CT imaging allowed for the detection of sites of urothelial carcinoma in all three patients (respectively a recurrent prostatic urethra lesion, a bladder mass and pelvic lymph nodes, and retroperitoneal lymph nodes and lung metastases), overall levels of radiotracer uptake were low. Consistent with this observation, IHC staining of tissue from one of the imaged patients demonstrated a low level of neovascularization and nearly absent PSMA expression.

## 4. Primary Brain Tumors

The most common primary brain tumors are gliomas, with grades I, II, and III usually progressing to a poor outcome over 2 to 10 years and the most aggressive grade IV (glioblastoma multiforme (GBM)) usually resulting in death within 2–3 years following diagnosis [22,23,24]. The current standard therapy of gliomas includes maximal safe surgical resection, followed by concurrent radiation (with temozolomide (TMZ), an oral alkylating chemotherapy agent in glioblastoma), and then adjuvant chemotherapy with TMZ in glioblastoma [22]. Extensive and complete surgical resection of gliomas is difficult because these tumors are frequently invasive, in the case of glioblastoma, and are often in eloquent areas of the brain, including areas that control speech, motor function, and the senses. Because of the high degree of invasiveness and the issue of location, radical resection of the primary tumor mass is often not curative, and infiltrating tumor cells invariably remain within the surrounding brain, later on leading to disease progression or recurrence [23,24]. At recurrence, a minority of patients are eligible for second surgery or reirradiation, based on appropriate patient selection. In temozolomide-pretreated patients, progression-free survival rates at 6 months of 20%–30% may be achieved with either nitrosoureas, temozolomide in various dosing regimens, or bevacizumab. Therapeutic targeting of the endothelium in gliomas is attractive as it may be exposed to a therapeutic agent much more readily than the tumor substance which is protected by the blood–brain barrier. As such, PSMA targeting in gliomas may offer a theranostic outlook for glioma patients.

Sasikumar et al. performed 68Ga-PSMA-HBED-CC PET/CT imaging in 10 patients with suspected recurrence on MRI of a previously treated glioblastoma [25]. The 68Ga-PSMA-HBED-CC PET scan was positive in nine patients, and subsequent histopathology proved it to be true recurrence. In the scan-negative case on MRI follow-up at nine months, no evidence of disease could be identified. The same authors compared results obtained using FDG PET and 68Ga-PSMA-HBED-CC PET imaging in five treated cases of glioblastoma with suspected recurrence; the findings from 68Ga-PSMA-HBED-CC PET/CT proved concordant with those from the FDG PET/CT scan [26]. However, when compared to FDG PET/CT, 68Ga-PSMA-HBED-CC PET/CT showed better visualization of the recurrent lesion (presence/absence) owing to its significantly high tumor-to-background ratio (TBR, mean TBR of 12.9 versus 0.96). Similar results were reported by Verma et al. and Salas Fragomeni et al. [27,28]. In a series of 10 glioma patients (7 GBM and 3 low-grade gliomas), Verma et al. found high FDG uptake in GBM, whereas the low-grade gliomas were not detected in the FDG study because of the fact that their activity was comparable to that of the physiological brain uptake [27]. Inversely, all of the gliomas were readily identified on the 68Ga-PSMA-HBED-CC PET/CT examinations, with the glioblastoma multiforme lesions presenting systematically with a higher SUVmax value (range 9.64–24.55) when compared to the low-grade gliomas (3.65–3.25). Salas Fragomeni et al. performed 18F-DCFPyL PET imaging in a series of three prospectively recruited patients suffering from high-grade glioma [28]. All three gliomas, probable GBM as defined by MRI imaging, demonstrated increased radiotracer uptake when compared with the background, similar to the results obtained using 68Ga-PSMA-HEBD-CC PET imaging. The SUVmax values, however, proved lower than those reported using 68Ga-PSMA PET imaging. The lower uptake when compared to that in 68Ga-PSMA-HBED-CC imaging may relate to differences in the biology of the gliomas studied or may be ascribed to the imaging agent.

## 5. Thyroid Carcinoma

Surgical thyroid removal is the definitive procedure for the management of differentiated thyroid carcinoma (DTC, papillary and follicular thyroid carcinoma) [29,30]. Approximately 4–6 weeks after surgical thyroid removal, patients may have radioactive iodine (RAI) therapy to detect and destroy any metastasis and residual tissue in the thyroid. If thyroid cancer recurs and the recurrence is radioiodine avid, the latter may be treated with radioiodine [29]. On the other hand, if patients present with RAI-refractory disease, which tends to be significantly worse than radioiodine-avid or non-progressive disease, they may be included in phase I, II, and III studies that are currently being conducted to evaluate the efficacy of new molecular-targeted drugs such as the tyrosine kinase inhibitors and re-differentiation drugs. The overall response rate of these drugs/therapies ranges between 0% and 53%, depending on whether the patients had been previously treated with these drugs, the performance status, and the extent of disease [30]. With the introduction of 68Ga-PMSA-HBED-CC PET imaging in patients suffering from prostate carcinoma, incidental uptake of 68Ga-PSMA-HBED-CC found in thyroid lesions has led a limited number of authors to explore in more detail the uptake of PSMA-targeting PET ligands by thyroid carcinoma neovasculature.

Lütje et al. studied six patients with RAI-refractory, FDG-positive metastasized DTC [31]. In five out of six patients, 68Ga-PSMA-HBED-CC PET imaging identified 41 lesions, all of which were confirmed by FDG PET/CT or conventional CT imaging. Whereas 68Ga-PSMA-HBED-CC PET imaging was fully concordant with FDG PET imaging in three patients, in the remaining two patients, only the most prominent lesions detected on FDG PET imaging were visualized using 68Ga-PSMA-HBED-CC PET imaging (SUVmax values ranged from 3.3 to 39.7). Finally, Verma et al. studied 10 patients suffering from metastasized DTC, 2 of which proved negative on iodine scintigraphy [32]. 68Ga-PSMA-HBED-CC PET imaging identified 30/32 lesions identified by all possible imaging techniques, whereas FDG PET imaging identified 23 lesions. Of the 30 lesions identified, 21 were localized to the bone. Furthermore, PSMA PET localized a lesion in each of the two RAI-refractory patients, similar to FDG PET imaging.

## 6. Breast Carcinoma

Breast carcinoma is the second leading cause of cancer-related death among women [33,34]. While novel therapies have improved the overall survival rate in breast carcinoma patients, treatment-related side effects and resistance warrant the need for new risk stratification and therapeutic targets. In this regard, PSMA expression on newly formed vessels in breast carcinoma may prove an interesting target for imaging and treatment.

Sathekge et al. studied 19 patients suffering from breast carcinoma in whom 81 tumor lesions were identified; out of these, 6 primary or recurrent lesions, 2 lymph nodes, and 5 metastases proved negative on 68Ga-PSMA-HBED-CC PET imaging, yielding an overall detection rate of 84% for 68-PSMA-HBED-CC PET/CT imaging [35]. The SUVmean values of distant metastases proved significantly higher when compared to those of primary or local recurrences or involved lymph nodes. Of interest, the 68Ga-PSMA-HEBD-CC SUVmean values of progesterone-receptor-positive lesions proved to be not significantly different from those obtained in progesterone-receptor-negative lesions.

## 7. Adenoid Cystic Carcinoma

Adenoid cystic carcinoma (ACC) accounts for approximately 20%–35% of all salivary gland malignancies, arising more often in the minor salivary glands. It is characterized by slow local progression, extensive perineural spread, and a tendency for delayed onset of distant metastases [36]. Following surgery, frequently followed by adjuvant radiation therapy, almost half of the patients develop slowly growing distant metastases within the first five years following diagnosis, significantly reducing patient prognosis; the reported five-year survival rates are 7%–32%. While the current guidelines consider FDG PET/CT imaging at initial presentation to assess disseminated disease, FDG uptake in ACC is lower than that in squamous cell carcinoma, and not all ACC show detectable uptake. Klein Nulent et al. performed 68Ga-PSMA-HBED-CC PET/CT imaging in nine ACC patients because of either suspected local recurrence or distant metastasis [37]. In addition, the authors performed IHC for PSMA on representative tumor samples, all of which proved positive. Furthermore, with the exception of one patient, all matched cases showed concordant positive PSMA expression between primary and recurrent cases. All nine 68Ga-PSMA-HBED-CC PET examinations clearly depicted tracer uptake in areas of the former primary tumor or localizations of distant metastasis. Three examinations showed tracer uptake in four new lesions suspected of being metastases; progression of formerly diagnosed metastases was seen in four patients.

## 8. Hepatocellular Carcinoma

The diagnosis of hepatocellular carcinoma (HCC), the most frequent primary liver malignancy, is challenged by the silent course of the disease, fluctuating serum alpha-fetoprotein levels, and inconclusive radiological findings [38]. Whereas FDG PET imaging has been shown to be of clinical value for detecting metastatic lymph nodes, distant metastases, and recurrent HCC, it is of limited value for the initial diagnosis of HCC. Accordingly, novel tracers with a higher sensitivity than that of FDG for primary HCC identification are of interest. In this regard, Kessler et al. assessed the potential role of 68Ga-PSMA-HBED-CC PET in a pilot study of seven patients (six newly diagnosed) suffering from HCC with 41 liver lesions, of which 37 were suspected to be tumoral, the remaining 4 being regenerative nodules [39]. Thirty-six of the 37 tumor lesions and none of the regenerative nodules showed tracer uptake, while only ten lesions were FDG avid. The one lesion that was negative on 68Ga-PSMA-HBED-CC PET also proved nonenhancing on contrast CT, whilst the remaining lesions were, and 68Ga-PSMA-HBED-CC uptake proved highly correlated with vascularity. Of interest, 68Ga-PSMA-HBED-CC PET/CT imaging identified unexpected extra-hepatic metastases in two out of the seven patients studied. More recently, Kuyumcu et al. reported on results obtained in 17 HCC patients who were referred for restaging of a known HCC and who underwent both FDG and 68Ga-PSMA-HBED-CC PET/CT imaging [40]. Whereas in two patients HCC uptake was both FDG and 68Ga-PSMA-HBED-CC PET negative, FDG uptake proved higher than that of the PSMA-targeting ligand (both visually and quantitatively (SUVmax)) in four patients (one of these patients being FDG negative), whereas in nine patients Ga-PSMA-HEBD-CC uptake by their HCC proved higher when compared to that of FDG (respectively negative in two patients).

## 9. Discussion

To date, large, well-designed studies addressing the role of 68GA-PSMA-HBED-CC or 18F-DCFPyl PET/CT imaging targeting PSMA expression on tumors other than prostate carcinoma are lacking. Nevertheless, the limited available data, mainly retrospective in nature, are promising and in line with available histological studies on PSMA expression in the various tumor types studied. Not surprisingly, the majority of the tumor types under study are those in which FDG, the most common tumor imaging agent for PET/CT imaging, has low sensitivity either due to high physiological uptake in the surrounding normal tissue, e.g., brain tumors; due to their indolent course reflected in a low glucose metabolism, e.g., adenoid cystic carcinoma; or to specific underlying molecular characteristics limiting their degree of FDG accumulation, e.g., overactivity of the glucose-6-phosphatase enzyme (removing 18F from the FDG molecule) in differentiated HCC.

Consistent with published data in pathology showing present yet intermediate expression of PSMA in the tumor neovasculature of non-clear cell RCC subtypes, only a small portion of sites of non-clear cell RCC were shown to take up the PSMA-targeting radiotracer 18F-DCFPyL, indicating that PSMA-based PET/CT imaging is likely not appropriate for imaging of these tumors. Inversely, in CRCC demonstrating high levels of PSMA expression, 68Ga-PSMA-HBED-CC and 18F-DCFPyl PET/CT imaging may have a clinically useful role in the evaluation of those patients presenting with indeterminate findings on conventional imaging, such as retroperitoneal and mediastinal lymph nodes measuring less than 1 cm in diameter [10]. Furthermore, the higher reported detection rate of metastatic lesions when using PSMA-targeting PET/CT imaging in CRCC as compared to conventional imaging suggests that PSMA-targeting PET/CT imaging may play a significant role in confirming the diagnosis as well as assessing the extent of oligometastatic CRCC, thereby significantly impacting the outcome for these patients [10]. As indicated by a recent systematic review, complete metastasectomy in oligometastatic CRCC patients is associated with a significant gain in median overall survival (36.5–142 months versus 8.4–27 months in those patients who undergo an incomplete metastasectomy) [41]. In urothelial carcinoma, the relatively scant expression of PSMA as evidenced from PSMA IHC studies is likely to limit the utility of PSMA-targeted PET imaging. In patients suffering from glioma, limited available data suggest that it may prove highly accurate for the purpose of detecting tumor recurrence, with the absence of PSMA-targeting PET ligand uptake in the normal brain parenchyma and rupture of the blood–brain barrier allowing facile accessibility of the ligand to its target resulting in high tumor-to-background ratios and straightforward visualization of the recurrence. A similar potential role for recurrence detection using PSMA PET imaging may also apply to adenoid cystic carcinoma of the head and neck. In patients suffering from thyroid carcinoma, limited available data suggest that PSMA PET imaging may prove of specific interest in the subsets of RAI-refractory and aggressive high-grade thyroid carcinoma as it tends to detect more metastatic RAI-resistant foci and proved more effective in recognizing brain lesions when compared to FDG PET/CT. In breast carcinoma, PSMA PET imaging demonstrated a considerable variation of PSMA expression in primary and metastatic breast carcinoma lesions from one patient to another as well as from one lesion to another within one patient, supporting the fact that it is a heterogeneous disease. Finally, limited available data suggest a potential role for PSMA-targeted PET imaging in the differential diagnosis and staging of HCC.

The high uptake of PSMA-targeting ligands by solid tumors other than prostate carcinomas, based on underlying overexpression of this marker on their neovasculature and the lack thereof on normal vessels as evidenced by histology, renders PSMA a highly interesting target for antiangiogenic therapy. First, PSMA-targeting agents such as, e.g., 177Lu-PSMA-targeting or PSMA-targeting nanoparticles containing chemotherapeutics may either selectively destroy tumor neovascularization or allow for a selective delivery of high doses of chemotherapeutics, overcoming tumor resistance whilst sparing normal tissues. Patients that could potentially benefit from this type of treatment, e.g., those with glioblastoma, could be selected based on their PSMA PET imaging results. Second, according the Jain hypothesis, treatment with anti-angiogenic agents such as bevicuzamb, which captures free human vascular endothelial growth factor-A (VEGF-A, are assumed to improve chemotherapy efficacy by transiently normalizing the leaky character of the tumor vessels, leading to more effective oxygen and drug delivery [42]. Our group previously showed in an animal model that when given outside this normalization window, chemotherapeutics proved ineffective [43]. Thus, suboptimal clinical results obtained using bevacizumab in combination with chemotherapy may have resulted from an inappropriate sequence in the timing of the administration of chemotherapeutics. As preclinical findings suggest that PSMA may be involved in the leaky character of the endothelium of newly formed vessels [44], PSMA-targeting PET/CT imaging may provide a potential tool for characterization of the “normalization window” which, in turn, may then allow for optimization of the efficacy of VEGF-targeting therapies such as bevacizumab.

## 10. Conclusions

PSMA expression in the tumor neovasculature has been established using IHC in a wide variety of tumors. Positron-emitting small molecules targeting PSMA have been developed for PET imaging, and their potential as an alternative imaging modality for staging and restaging in tumor types where FDG PET/CT imaging has low diagnostic accuracy is currently being explored. Preliminary, mainly retrospective studies are encouraging, with evidence of improved diagnostic sensitivity and specificity in CRCC, glioma, and HCC, leading to a change in patient management in several patients. The results published thus far warrant confirmation by larger prospective studies additionally assessing the longitudinal impact on patient outcomes.
